# O024: New holistic approach to determine the infection risk profile of a hospital; visualised in a easy-to-read plot

**DOI:** 10.1186/2047-2994-2-S1-O24

**Published:** 2013-06-20

**Authors:** I Willemsen, J Kluytmans

**Affiliations:** 1 Department for Medical Microbiology and Infection control, VUmc Medical Center, Amsterdam, The Netherlands; 2 Laboratory of Microbiology and Infection control, Amphia hospital, Breda, The Netherlands

## Introduction

Prevalence surveys are common tools to determine the prevalence of- and determinants for Healthcare Associated Infections (HAI). However this only incorporates patient-related variables.

## Objectives

We developed a new method, the “infection risk scan”, which includes outcome variables, patient-related variables as well as ward-related variables. This should provide a holistic view on the infection risk profile of a ward or a hospital.

## Methods

Two outcome variables were investigated, prevalence of healthcare associated infections (HAI) and rectal carriage of Extended Spectrum B-Lactamase (ESBL) producing bacteria. Two patient-related risk variables, use of indwelling medical devices and antimicrobial therapy, and two ward-related variables, environmental contamination and hand hygiene non-compliance (according to the WHO guideline). Results of all investigated variables were categorised as low risk, medium risk and high risk, based on the literature or expert opinion, and presented in a spider-plot.

The infection risk scan was performed in 4 different general nursing wards.

## Results

Large differences were found in outcome variables and risk factors, with a distribution across all 3 risk categories (low, medium and high). This resulted in different risk-plots for the different wards. Handhygiene non-compliance and the environmental contamination were a cause of concern in all wards. Prevalence of ESBL carriage was low in all wards, and the ESBL isolates were genotypically not related.

## Conclusion

In conclusion, the infection risk plot demonstrated substantial differentiation. The plot gives an overview that can easily be understood by the healthcare workers and managers. The problem areas are shown at a glance. Based on the findings a tailor made, targeted quality improvement project can be executed and the results can be measured in a repeated measurement. This makes the infection risk scan a management tool that can be used to determine the scope and focus of an infection control program.

## Disclosure of interest

None declared.

